# Study on the Enhancement of Mechanical Properties and Electromagnetic Performance of Imidazolium Ionogels by Doping with Magnetic Triiron Tetraoxide Nanoparticles

**DOI:** 10.3390/polym18131614

**Published:** 2026-06-29

**Authors:** Xueqi Zhao, Zhanrong Zhou, Peijia Ding, Yang Gao, Xingyu Xie, Hongfu Qiang, Jian Hu

**Affiliations:** 1 National Key Laboratory of Solid Rocket Propulsion, Rocket Force University of Engineering, Xi’an 710025, China; 18729396064@163.com (X.Z.);; 2 State Key Lab for Strength and Vibration of Mechanical Structures, International Center for Applied Mechanics, Department of Engineering Mechanics, Xi’an Jiaotong University, Xi’an 710049, China

**Keywords:** ionogels, magnetic nanocomposites, mechanical properties, electromagnetic properties, dielectric relaxation

## Abstract

Ionogels combining ionic liquids with polymer networks show promise for flexible electronics, but their mechanical and functional performance often needs enhancement. Here, we report a series of magnetic nanocomposite ionogels fabricated by doping triiron tetraoxid (Fe_3_O_4_) nanoparticles into a [C_2_mim]^+^[EtSO_4_]^−^-dispersed cross-linked PAA matrix. The effect of PAA content (10–20 wt%) on the optical, mechanical, and dielectric properties of pure imidazolium ionogels was first investigated. Increasing PAA concentration enhanced tensile strength (up to ~0.7 MPa) and compressive modulus (~0.65 MPa) while reducing optical transmittance; dielectric relaxation peaks around 6–8 GHz were observed, with the 15 wt% sample showing the highest permittivity. Subsequently, Fe_3_O_4_ nanoparticles (0–20 wt%) were incorporated into the 10 wt% PAA ionogel. The resulting magnetic ionogels exhibited reduced tensile strength, but significantly increased elongation (up to ~12 strain), indicating network softening. Magnetic hysteresis measurements confirmed superparamagnetic behavior with saturation magnetization reaching ~2.5 emu/g at 20 wt% Fe_3_O_4_ loading. This work demonstrates a facile strategy to simultaneously tune mechanical, dielectric, and magnetic properties in imidazolium ionogels, providing guidelines for designing soft multifunctional materials for microwave absorption, magnetic actuation, and flexible sensor applications.

## 1. Introduction

Ionogels are hybrid materials composed of ionic liquids (ILs) immobilized within three-dimensional cross-linked polymer or inorganic networks [1,2,3]. They uniquely combine the advantageous properties of ILs—including high ionic conductivity, negligible volatility, wide electrochemical stability window, and non-flammability—with the mechanical integrity, flexibility, and processability of solid polymer networks [4,5,6]. Among various ILs, imidazolium-based ones, such as 1-ethyl-3-methylimidazolium ethyl sulfate ([C_2_mim]^+^[EtSO_4_]^−^), u+22are particularly attractive due to their low viscosity, high conductivity, and good biocompatibility [7,8,9,10]. Poly(acrylic acid) (PAA) is a widely used polymer matrix for imidazolium ionogels, offering excellent film-forming ability and tunable mechanical properties via cross-linking density [11,12,13]. These attributes make ionogels promising candidates for flexible electronics, solid-state electrolytes, sensors, actuators, and wearable devices, with typical ionic conductivity of 10^−3^–10^−2^ S/cm and elongations exceeding 1000% [8,14,15]. However, the mechanical strength of pure ionogels often limits their practical applications, motivating strategies such as nanoparticle reinforcement [12,16,17].

Incorporating functional nanoparticles (NPs) into ionogel matrices has emerged as an effective strategy to overcome these limitations [11]. Magnetic NPs, especially triiron tetraoxide (Fe_3_O_4_), are of particular interest due to their inherent superparamagnetism, high saturation magnetization, biocompatibility, and ease of surface functionalization [18,19,20]. In the context of electromagnetic wave modulation, Wu et al. [21] demonstrated that the dielectric properties of hydrogel, organogel, and ionogel absorbers are highly correlated with the polarity, ionic conductivity, and non-covalent interactions of the built-in liquids. Subsequently, Dong et al. [22] developed an adaptive ionic liquid polymer microwave modulation surface with reprogrammable dielectric properties, while Wu et al. [23] further enhanced ionogel-based microwave absorption through molecular structure engineering and microphase separation synergy. Wu et al. [24] also reported a soft giant magnetoimpedance ionogel (GelGMI) by dispersing electrostatically self-assembled ferromagnetic domains in a soft ionogel matrix, achieving record sensitivity, stretchability (>1000%), neuromorphic temporal accumulation, and self-healing capability. Zhang et al. [25] constructed a deformation-engineered multidimensional magnetoelectric coupling gel network that integrates magnetic nanoparticles, dielectric components, and conductive carbon frameworks, producing mechanically tunable electromagnetic wave absorption. When embedded in a soft ionogel network, Fe_3_O_4_ NPs can both enhance mechanical properties through reinforcement and impart electromagnetic responsiveness, enabling remote actuation, magnetic field sensing, and tunable microwave absorption [26,27].

It is important to distinguish our ionogel system from conventional aqueous magnetic PAA hydrogels, which have been extensively explored for biomedical applications such as drug delivery and tissue engineering [26,28]. Our ionogel system offers several distinct advantages: (i) the use of [C_2_mim]^+^[EtSO_4_]^−^ ionic liquid as the solvent medium imparts non-volatility, a wider electrochemical window, and better thermal stability compared to water-based hydrogels; (ii) ionogels exhibit higher ionic conductivity (10^−3^–10^−2^ S/cm) due to the high concentration of free ions; (iii) unlike hydrogels that suffer from water evaporation and freezing, ionogels maintain stable performance across wide temperature and humidity ranges; and (iv) the presence of mobile ions in ionogels gives rise to unique dielectric relaxation phenomena in the GHz range, which is not observed in conventional hydrogels [29]. These distinctions make ionogels better suited for flexible electronics, microwave absorbers, and soft actuators [3,30].

Despite these remarkable advances, key questions regarding the effect of nanoparticle content on the tensile and compressive behavior, static magnetic properties, and broadband dielectric response (0–18 GHz) of imidazolium ionogels have yet to be addressed [22,23]. Specifically, quantitative relationships between Fe_3_O_4_ nanoparticle loading and the coupled mechanical–electromagnetic performance have not been established for the [C_2_mim]^+^[EtSO_4_]^−^/PAA system. A systematic study focusing on the synergistic enhancement of both mechanical and electromagnetic properties in imidazolium ionogels through controlled doping with Fe_3_O_4_ nanoparticles remains underexplored [1,31,32].

Therefore, this study aims to fabricate a series of magnetic nanocomposite ionogels by embedding PAA-coated Fe_3_O_4_ NPs into a [C_2_mim]^+^[EtSO_4_]^−^-dispersed cross-linked PAA network. First, we investigate the influence of PAA concentration (10–20 wt%) on the optical, mechanical, and dielectric properties of the base ionogel. Subsequently, we incorporate varying weight fractions of Fe_3_O_4_ NPs (0–20 wt%) to systematically evaluate their impact on morphology, tensile and compressive mechanical performance, static magnetic properties (hysteresis loops), and high-frequency dielectric behavior. The results establish structure-property relationships that provide rational design guidelines for advanced magnetic ionogels with tailored mechanical and electromagnetic performance, catering to next-generation flexible and responsive devices such as microwave absorbers, magnetic actuators, and soft sensors.

## 2. Materials and Methods

### 2.1. Materials

The following chemicals were obtained from commercial suppliers and were used without further purification: Iron (II) chloride tetrahydrate (FeCl_2_·4H_2_O, Sigma-Aldrich, Shanghai, China, 44939), Iron (III) chloride hexahydrate (FeCl_3_·6H_2_O, Sigma-Aldrich, Shanghai, China, F2877), Sodium hydroxide (NaOH, AR, Tianjin Xinbote Chemical Co., Ltd., Tianjin, China), Poly(acrylic acid) solution (PAA, Aladdin, Shanghai, China, P299190), 1-Ethyl-3-methylimidazolium ethyl sulfate ([C_2_mim]^+^[EtSO_4_]^−^, Macklin Inc., Shanghai, China, E809290), Acrylic acid (AA, Aladdin, Shanghai, China, A103526), Ammonium persulfate (APS, Aladdin, Shanghai, China, A112450), and N,N′-Methylenebis(acrylamide) (MBAA, Aladdin, Shanghai, China, M128783).

### 2.2. Synthesis of Magnetic Nanoparticles (NPs)

**Figure 1:**
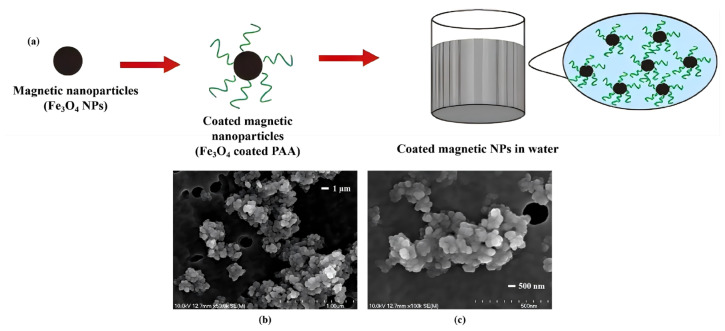
Design and fabrication of magnetic nanoparticles (NPs). (**a**) Schematic illustration of PAA-coated Fe_3_O_4_ NPs in water. (**b**) SEM image of 1 μm and (**c**) 500 nm.

Fe_3_O_4_ NPs were synthesized via coprecipitation [16,33]. FeCl_2_·4H_2_O (0.0436 mol) and FeCl_3_·6H_2_O (0.08 mol) (the molar ratio of Fe(II) to Fe(III) is 0.55:1) were dissolved in 188 mL (0.08 mol) (Fe(II): Fe(III) molar ratio = 0.55:1) were dissolved in 188 mL deionized water and filtered. NaOH solution (5.0 M, 100 mL) was added to precipitate black magnetic NPs (pH = 14). The mixture was heated at 60 °C for 30 min with vigorous stirring, then at 80 °C for 1 h under N_2_ with slow stirring, yielding a ferrofluid. To coat NPs with PAA, 0.16 mol PAA solution was added to 300 mL of the NP suspension (final molar ratio Fe(II): Fe(III): PAA = 0.55:1:2). The mixture was stirred at 90 °C for 4 h. The product (PAA-coated Fe_3_O_4_) was magnetically separated and washed with water to neutral pH. The final suspension was stored at room temperature. A field-emission scanning electron microscopy (FE-SEM, Hitachi S-4700, Hitachi High-Technologies Corporation, Tokyo, Japan) showed the coated NPs were spherical, with diameters around 500 nm (Figure 1).

### 2.3. Preparation of Magnetic NPs/Ionic Liquid Nanofluid

The magnetic NP suspension was transferred to a 250 mL polypropylene bottle. A magnet placed beneath the bottle collected the NPs. After layer separation, the clear supernatant was removed, yielding a concentrated NP paste. To this paste, 50 g of [C_2_mim]^+^[EtSO_4_]^−^ (recorded as m_IL_) was added. The mixture was stirred at 30–60 °C to form a uniform three-phase suspension (water/ionic liquid/NPs). It was then dried under gradient vacuum (40–60 °C, pressure < −0.08 MPa) until mass stabilization (change rate < 0.5%), with the final mass recorded as mtotal. This process allowed ionic liquid to replace the hydration layer around NPs via interaction with PAA groups. Finally, the dried material was gently stirred at 50–70 °C for 10–30 min, producing a homogeneous, stable, and flowable concentrated nanofluid. The NP mass percentage in the total suspension (*W*_NPs_) was calculated using Formula (1).

(1) $$w_{NPs}=\frac{m_{total}-m_{IL}}{m_{total}}\times 100\%=\frac{m_{NPs}}{m_{total}}\times 100\%$$

### 2.4. Synthesis of the Imidazolium Ionogels

A homogeneous solution was prepared by mixing acrylic acid (AA), the cross-linker MBAA, and the initiator APS in [C_2_mim]^+^[EtSO_4_]^−^ ionic liquid. The AA concentrations were 10, 15, and 20 wt% relative to the total ionogel mass. The concentrations of MBAA and APS were 0.1 wt% and 1 wt%, respectively, relative to the AA mass. The solution was transferred to a glass mold and reacted at 45 °C for 6 h to complete gelation [28]. Under these conditions, the polymerization is known to achieve high conversion, as confirmed by the mechanical integrity and stability of the resulting ionogels [9]. The resulting pure PAA ionogels were cut into desired shapes for testing.

### 2.5. Synthesis of the Magnetic NPs/Imidazolium Ionogels

**Figure 2:**
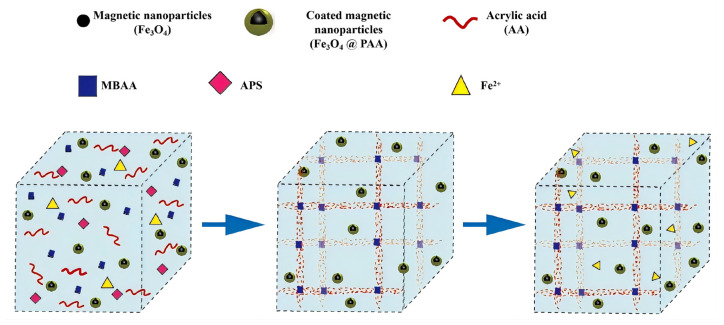
Schematic illustration of the composite structure of the magnetic NP/imidazolium ionogel. The magnetic nanoparticles are suspended within a cross-linked poly(acrylic acid) network swelled by the ionic liquid.

The concentrated nanofluid (*W*_NPs_)) was used to synthesize magnetic ionogels. AA, MBAA, APS, and the nanofluid were mixed in [C_2_mim]^+^[EtSO_4_]^−^. The AA concentration was fixed at 10 wt% of the total ionogel mass. The MBAA and APS concentrations were 0.1 wt% and 1 wt%, respectively, relative to AA. The magnetic NP content was varied at 0, 5, 10, and 15 wt% of the total ionogel mass. All components were stirred into a homogeneous solution, transferred to a glass mold, and reacted at 45 °C for 6 h. The magnetic ionogels were synthesized via in situ free radical polymerization within the ionic liquid (Figure 2). The resulting magnetic NP/imidazolium ionogels were cut into desired shapes for testing.

## 3. The Pure Imidazolium Ionogels

### 3.1. Optical Properties of the Imidazolium Ionogels

**Figure 3:**
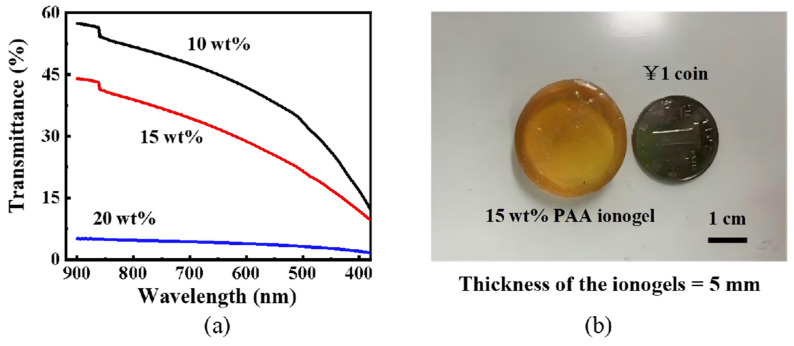
(**a**) UV-Vis transmittance spectra of pure PAA ionogels with different weight fractions (10 wt%, 15 wt%, 20 wt%) in the wavelength range of 400–900 nm; (**b**) digital photograph of a pure PAA ionogel with a thickness of 5 mm, compared with a ¥1 coin to illustrate its macroscopic morphology and size.

Figure 3 presents the optical transmittance and macroscopic morphology of the PAA ionogels. In Figure 3a, the UV-Vis transmittance spectra demonstrate that within the 400–900 nm wavelength range, the transmittance of the ionogels decreases significantly with increasing PAA mass fraction. The 10 wt% sample exhibits a transmittance of approximately 58% at 900 nm, which gradually declines to about 15% at 400 nm. The 15 wt% sample shows intermediate transmittance, ranging between 10% and 45% across the entire spectrum. The 20 wt% sample has the lowest transmittance, remaining mostly below 5% and appearing nearly opaque. All samples display a trend of decreasing transmittance with shorter wavelengths, attributed to enhanced light scattering and absorption caused by molecular aggregation or phase separation within the system due to higher PAA content. Figure 3b shows a macroscopic photograph of a pure PAA ionogel with a thickness of 5 mm. The sample appears as a semi-transparent, amber-colored disk with excellent mechanical integrity, maintaining its macroscopic shape stably. Its dimensions are comparable to a 1-yuan coin (approximately 20 mm in diameter), highlighting its superior film-forming properties and processability. Regarding optical transmittance, increasing PAA concentration from 10 wt% to 20 wt% significantly reduces optical transmittance across the entire 400–900 nm wavelength range. Specifically, at 900 nm, transmittance decreases from ~58% (10 wt%) to ~45% (15 wt%) to below 5% (20 wt%); at 400 nm, transmittance decreases from ~15% (10 wt%) to ~10% (15 wt%) to below 5% (20 wt%). The 20 wt% sample appears nearly opaque. This reduction is attributed to enhanced light scattering and absorption caused by increased molecular aggregation and microphase separation within the polymer network at higher PAA loadings.

### 3.2. Mechanical Properties of the Imidazolium Ionogels

**Figure 4:**
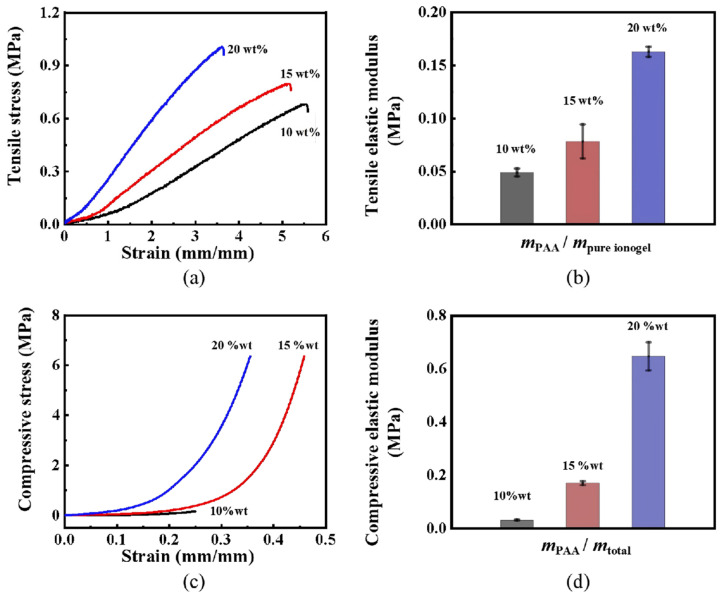
(**a**) Tensile stress–strain curves of PAA ionogels with different PAA weight fractions (10 wt%, 15 wt%, 20 wt%); (**b**) corresponding tensile elastic moduli of PAA ionogels, plotted as a function of the mass ratio of PAA to pure ionogel; (**c**) compressive stress–strain curves of PAA ionogels with different PAA weight fractions (10 wt%, 15 wt%, 20 wt%); (**d**) corresponding compressive elastic moduli of PAA ionogels, plotted as a function of the mass ratio of PAA to total mass.

Figure 4 illustrates the mechanical properties of PAA ionogels with varying PAA weight fractions under tensile and compressive loading. As shown in the tensile stress–strain curves (a) and corresponding elastic modulus bar chart (b), both tensile strength and tensile elastic modulus increase with higher PAA content: the 20 wt% sample exhibits the highest tensile stress (~0.7 MPa) and tensile elastic modulus (~0.17 MPa), followed by the 15 wt% sample (~0.8 MPa tensile stress, ~0.08 MPa modulus) and the 10 wt% sample (~0.7 MPa tensile stress, ~0.05 MPa modulus). Similarly, the compressive stress–strain curves (c) and compressive elastic modulus bar chart (d) reveal that compressive performance is also enhanced with increasing PAA content: the 20 wt% sample achieves the highest compressive stress (~7 MPa at 0.35 strain) and compressive elastic modulus (~0.65 MPa), while the 15 wt% sample shows moderate compressive stress (~7 MPa at 0.45 strain) and modulus (~0.18 MPa), and the 10 wt% sample displays the lowest compressive stress and modulus (nearly negligible across the tested strain range). These results demonstrate that increasing the PAA weight fraction effectively reinforces the mechanical strength and stiffness of PAA ionogels under both tensile and compressive conditions, which is attributed to the enhanced intermolecular interactions and cross-linking density within the ionogel network at higher PAA loading [9,13].

### 3.3. Dielectric Properties of the Imidazolium Ionogels

**Figure 5:**
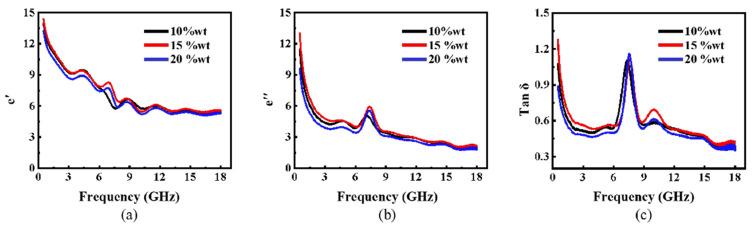
(**a**) Frequency dependence of the real part of permittivity (ε′), (**b**) the imaginary part of permittivity (ε″), and (**c**) the dielectric loss tangent (tan δ) for PAA ionogels with different PAA weight fractions (10 wt%, 15 wt%, 20 wt%) in the frequency range of 0–18 GHz.

Figure 5 presents the dielectric properties of PAA ionogels with varying PAA weight fractions across the 0–18 GHz frequency range. As shown in plot (a), the real permittivity (ε′) of all samples decreases with increasing frequency, exhibiting a stepwise decline followed by stabilization at ~5–6 above 12 GHz, with the 15 wt% sample consistently showing slightly higher ε′ values than the 10 wt% and 20 wt% samples. In plot (b), the imaginary permittivity (ε″) follows a similar decreasing trend with frequency, accompanied by a distinct peak around 6–7 GHz for all samples, indicating a dielectric relaxation process, and the 15 wt% sample displays the highest ε″ among the three formulations. For the dielectric loss tangent (tan δ) in plot (c), all samples exhibit a prominent relaxation peak around 7–8 GHz, with the 20 wt% sample reaching the highest peak value (~1.15), followed by the 15 wt% and 10 wt% samples, while tan δ values stabilize at ~0.3–0.5 at frequencies above 12 GHz across all formulations. These results reveal that PAA weight fraction influences both the dielectric constant and loss characteristics of the ionogels, with relaxation peaks attributed to dipole orientation and interfacial polarization within the PAA-ionogel network, and the frequency-dependent behavior aligns with typical dielectric relaxation mechanisms in polymer-based ionic materials [29].

## 4. Properties of the Magnetic NPs/Imidazolium Ionogels

**Figure 6:**
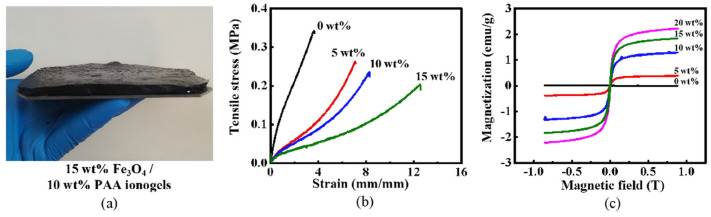
(**a**) Digital photograph of a 15 wt% Fe_3_O_4_/10 wt% PAA ionogel, demonstrating its flexible macroscopic morphology; (**b**) tensile stress–strain curves of PAA ionogels with different Fe_3_O_4_ weight fractions (0 wt%, 5 wt%, 10 wt%, 15 wt%); (**c**) magnetic hysteresis loops of PAA ionogels with different Fe_3_O_4_ weight fractions (0 wt%, 5 wt%, 10 wt%, 15 wt%).

Figure 6 illustrates the morphology, mechanical properties, and magnetic performance of Fe_3_O_4_/PAA ionogels with varying Fe_3_O_4_ contents. The photograph in (a) shows that the 15 wt% Fe_3_O_4_/10 wt% PAA ionogel exhibits good flexibility and processability, maintaining a stable macroscopic shape. As expected, the incorporation of Fe_3_O_4_ nanoparticles renders the ionogels visually opaque due to strong light absorption, consistent with the dark appearance.

From the tensile stress–strain curves in (b), it is clear that both tensile strength and strain at break decrease with increasing Fe_3_O_4_ content: the 0 wt% Fe_3_O_4_ sample achieves the highest tensile stress (~0.35 MPa) at ~4 strain, while the 15 wt% Fe_3_O_4_ sample shows the lowest tensile stress (~0.2 MPa) but the largest elongation (~12 strain), indicating that Fe_3_O_4_ incorporation softens the ionogel network and reduces mechanical strength while extending ductility [34,35]. The trade-off between strength and extensibility is consistent with observations in other nanoparticle-reinforced gel systems, where nanoparticles can act as physical defects that reduce stress-bearing capacity while enabling greater chain mobility. The magnetic hysteresis loops in (c) reveal that the magnetization of the ionogels increases monotonically with Fe_3_O_4_ content, with the 20 wt% Fe_3_O_4_ sample reaching the highest saturation magnetization (~2.5 emu/g), and all Fe_3_O_4_-loaded samples exhibit typical superparamagnetic behavior with negligible coercivity and remanence, confirming the successful introduction of magnetic functionality into the PAA ionogels without compromising their processability [36].

The introduction of Fe_3_O_4_ nanoparticles imparts multiple functional benefits to the PAA-based ionogels. First, Fe_3_O_4_ NPs confer superparamagnetic behavior (saturation magnetization ~2.5 emu/g at 20 wt% loading), enabling magnetic field responsiveness, remote actuation, and magnetic sensing. Second, while Fe_3_O_4_ incorporation reduces tensile strength (from ~0.35 to ~0.2 MPa), it dramatically increases elongation (from ~4 to ~12 strain), enabling larger deformation without fracture. Third, the combination of dielectric loss (from the ionogel matrix) and magnetic loss (from Fe_3_O_4_ nanoparticles) can potentially enhance microwave absorption through improved impedance matching and multiple loss mechanisms. Fourth, Fe_3_O_4_-doped ionogels can respond to both electric fields (via ionic conduction) and magnetic fields (via nanoparticle magnetization), enabling applications in soft robotics, magnetic actuation, and multifunctional sensors.

## 5. Conclusions

In this study, a series of magnetic nanocomposite ionogels were successfully synthesized by embedding PAA-coated Fe_3_O_4_ nanoparticles into [C_2_mim]^+^[EtSO_4_]^−^-swollen cross-linked PAA networks. Increasing PAA content (10–20 wt%) enhanced tensile strength (~0.7 MPa) and compressive modulus (~0.65 MPa) while reducing optical transmittance and inducing dielectric relaxation peaks around 6–8 GHz. Incorporating Fe_3_O_4_ nanoparticles (0–20 wt%) softened the network, decreasing tensile strength (from ~0.35 to ~0.2 MPa) but dramatically increasing elongation (from ~4 to ~12 strain), and conferred superparamagnetic behavior, with saturation magnetization reaching ~2.5 emu/g at 20 wt% loading. These structure-property relationships provide a rational design strategy for tailoring mechanical and electromagnetic performance in soft multifunctional ionogels for applications such as flexible electronics, microwave absorption, and magnetic actuation.

## Data Availability

The raw data supporting the conclusions of this article will be made available by the authors upon request.
